# Partial Amino Acid Metabolism and Glutamine Synthesis as the Ammonia Defensive Strategies During Aerial Exposure in Chinese Loach *Paramisgurnus dabryanus*

**DOI:** 10.3389/fphys.2019.00014

**Published:** 2019-01-29

**Authors:** Yun-Long Zhang, Guang-Yi Wang, Zi-Han Zhang, Yun-Yi Xie, Hui Jin, Zhao-Ran Dong

**Affiliations:** College of Animal Science and Technology, Anhui Agricultural University, Hefei, China

**Keywords:** *Paramisgurnus dabryanus*, aerial exposure, amino acid metabolism, glutamine synthesis, ammonia defensive strategy

## Abstract

The *Paramisgurnus dabryanus* was exposed to air to assess the changes in plasma, liver and muscle free amino acid (FAA) contents. The FAA concentrations in plasma, liver and muscle of *P. dabryanus* were significantly affected by aerial exposure (*P* < 0.05). After 12 h of aerial exposure, the plasma glutamate contents increased significantly (*P* < 0.05) and reached peak value at 24 h of air exposure. With increasing air exposure time, the plasma alanine contents increased significantly and more dramatically than the control values (*P* < 0.05). From 24 to 48 h of aerial exposure, the liver free glutamate contents increased significantly and reached the peak value at 48 h of air exposure (*P* < 0.05). The liver free alanine contents in air exposure group were markedly higher than these values in the control group (*P* < 0.05). After 72 h of air exposure, the muscle free glutamate contents increased markedly (*P* < 0.05) and were significantly higher than the control values (*P* < 0.05). The muscle free alanine contents remained at constant values during the first 12 h of aerial exposure (*P* > 0.05), thereafter, these concentrations increased significantly until the end of experiment (*P* < 0.05). Our results showed that glutamate and NH_4_^+^ could be used to synthesize glutamine via glutamine synthetase to convert internal ammonia into non-toxic glutamine in *P. dabryanus* during air exposure. Furthermore, the *P. dabryanus* could catabolize several certain amino acids, leading alanine form to reduce endogenous ammonia production. The decrease in tissue free glutamate, arginine and proline in *P. dabryanus* indicated that these certain amino acids should be the starting substrate to be converted to alanine and energy.

## Introduction

Ammonia is toxic to aquatic animals (Zhang et al., 2017c) causing a decrease in growth and food intake (Naqvi et al., 2007; Paust et al., 2011), tissue damage (Rodrigues et al., 2011), gill morphological change (Dong et al., 2013), gill remodeling (Sinha et al., 2014), cell apoptosis and oxidative stress (Cheng et al., 2015; Pinto et al., 2016; Zhang et al., 2018). In teleosts, endogenous ammonia is mainly produced through amino acid catabolism and mainly excreted across the gills in the surrounded water (Ip and Chew, 2010; Chew and Ip, 2014). Moreover, external ammonia builds up in the surrounding water and reaches levels that can reverse the normal NH_3_ partial pressure gradient, causing ammonia excretion in fish to be inhibited and/or net uptake of ammonia from the environment to occur (Sinha et al., 2013). When the endogenous ammonia content reaches a considerable level, it has a lethal effect on most fish species.

In teleost, endogenous ammonia concentrations should be maintained at a low level to avoid the strong toxicity in general (Ip and Chew, 2010; Chew and Ip, 2014). However, some air-breathing fish, like *Paramisgurnus dabryanus* (Zhang et al., 2017b), *Misgurnus anguillicaudatus* (Chew et al., 2001; Tsui et al., 2004), *Monopterus albus* (Ip et al., 2004), *Boleophthalmus boddaerti*, and *Periophthalmodon schlosseri* (Ip et al., 2005), can accumulate quite high levels of endogenous ammonia. For these species, several ammonia detoxification strategies have been demonstrated to help deal with excessive endogenous ammonia loading, such as less toxic glutamine and urea synthesis (Sanderson et al., 2010; Loong et al., 2012), active NH_4_^+^ excretion (Tay et al., 2006), ammonia detoxification improved by Rhesus glycoproteins and aquaporins (Edwards et al., 2015; Chng et al., 2016), lowering of ambient pH (Chew et al., 2003a), NH_3_ volatilization and alkalization of body surface (Tsui et al., 2004), reduction in body ammonia production (Loong et al., 2012) and certain types of amino acid catabolism leading to alanine form (Sinha et al., 2013).

In general, inhibition of proteolysis and amino acid catabolism may be an effective ammonia tolerance strategy in fish because of the reduction of internal ammonia production (Chew and Ip, 2014). Nevertheless, the physiological processes of amino acid catabolism as the energy source to deal with stress situation is also inhibited due to the inhibition of proteolysis and amino acid catabolism. Thus, some fish species had to modify this strategy, they catabolized certain amino acids (e.g., glutamate, arginine and proline) leading to alanine form and produced ATP (Tsui et al., 2004). In fact, a series of transamination is catabolized to alanine without releasing ammonia. Consequently, partial amino acid catabolism leading to alanine form may be an effective ammonia defensive strategy. This adaptive ammonia tolerance strategy have been confirmed in several teleosts, such as *M. anguillicaudatus* (Chew et al., 2001; Tsui et al., 2004), *Channa asiatica* (Chew et al., 2003b) and *Cyprinus carpio* (Sinha et al., 2013).

The Chinese loach (*P. dabryanus*) distributes widespreadly in eastern Asian and is widely cultured in Asian countries, especially in China (Zhang et al., 2015a,b). According to China fishery statistical yearbook, the quantity of Chinese loach in 2016 was 400,209 tons (including *M. anguillicaudatus*). It can breathe air directly using the post-intestine as the accessory respiratory organ (Zhang et al., 2016). During periodical drought, *P. dabryanus* could survive for a few months via air breathing in the wet mud. Furthermore, the previous study have provided that *P. dabryanus* could accumulate quite high level of endogenous ammonia during air exposure (Zhang et al., 2017b). In this condition, excretion of endogenous ammonia into environment would be hindered with the largest extent. Interestingly, how *P. dabryanus* deal with the internal ammonia accumulation during the aerial exposure? The free amino acid (FAA) contents in plasma, liver and muscle of *P. dabryanus* have been determined during various period of air exposure, therefore, the aim of the present study is to verify the hypothesis that alanine synthesis through certain amino acid catabolism may be an effective ammonia detoxification strategy in *P. dabryanus* during aerial exposure.

## Materials and Methods

### Ethics Statement

This study was carried out in accordance with the principles of the Basel Declaration and recommendations of the Guide for the Care and Use of Laboratory Animals, the Animal Care Committee of Anhui Agricultural University (Hefei, China). The protocol was approved by the Animal Care Committee of Anhui Agricultural University.

### Experimental Fish

The experimental *P. dabryanus* (20–29 g) were obtained from a wet-market in Hefei. The fish were transferred to laboratory and reared in five 60 L tanks (30–50 individuals per tank) with 50 L dechlorinated water for 7 days at 25.5 ± 1.0°C before experimentation. During the rearing period, 30% water was changed daily and the water was aerated by 3 air stones. Fish were fed with a commercial compound diets (crude protein 35%, crude lipid 7%) twice daily. Fish were fasted for 24 h before the experiment and were unfed during the experimental period.

### Aerial Exposure

Four fish exposed to aerial condition were placed in plastic rounded boxes (diameter 35.5 cm, height 15.5 cm) containing a thin film of fresh water (100 ml) at 24 ± 1°C for various periods (0, 12, 24, 48, 72 or 96 h). The fresh water in the boxes was totally exchanged every 8 h to ensure the humidity for the entire period of terrestrial exposure. Fish submerged into 10 L dechlorinated water for various periods (0, 12, 24, 48, 72 or 96 h) served as controls. A total of 18 tanks were used for air exposure and there were three replicated tanks with 4 fish in each exposure periods.

### Sample Collection

At the end of each exposure period (0, 12, 24, 48, 72 or 96 h), all the fish were anaesthetized with 3-aminobenzoic acid ethyl ester (MS-222). Blood was quickly drawn from caudal vessels with heparinized syringes and then blood was centrifuged at 4,000 × g for 30 min at 4°C for collecting the plasma. Then, all the four fish in each replicate at each exposure period (0, 12, 24, 48, 72 or 96 h) were killed by a blow to the head. Then the fish were dissected to collect the liver and dorsal white muscle, samples were pooled from the four fish in each replicate and stored at –80°C in an ultra-low temperature freezer (Meiling, China) until biochemical assay. The experiments performed on animals, the animal care, and all protocols strictly followed the guidelines of the Anhui Agricultural University for the care and utilization of laboratory animals.

### Determination of Free Amino Acids

For plasma, 0.5 ml sample was collected and 2.5 ml 4% sulfosalicylic acid was added. Then, the mixture was centrifuged at 14,000 rpm for 30 min at 4°C. The resultant supernatant was carefully collected for the determination of FAA content. For liver and muscle, the samples were dried to a constant weight at 105°C. 0.5 g dry sample was weighed and ground using a mortar. 5 ml 4% sulfosalicylic acid was added and the mixture was centrifuged at 14,000 rpm for 30 min at 4°C. The resultant supernatant was carefully collected for the determination of FAA content. For the determination of FAA, the collected supernatant was passed through two Millipore micro filters (0.22 μm pore size) before assaying FAAs level. FAA contents was determined by using a Hitachi L-8900 automatic amino acid analyzer (Hitachi, Japan), equipped with an ion exchange resin column (4.6 mm × 60 mm).

### Statistical Analysis

Values of the measured variables are expressed as mean ± standard deviation. The variance homogeneity of the data was performed using Levene’s test. Data were compared by one-way ANOVA followed by Duncan’s test when significant differences were found at 0.05 level. The *t*-test was performed to compare the significance between aerial exposure and control at the same exposure time. Statistics were performed using SPSS 18.0 software (SPSS Inc., Chicago, IL, United States).

## Results

### Plasma FAA Contents

**FIGURE 1:**
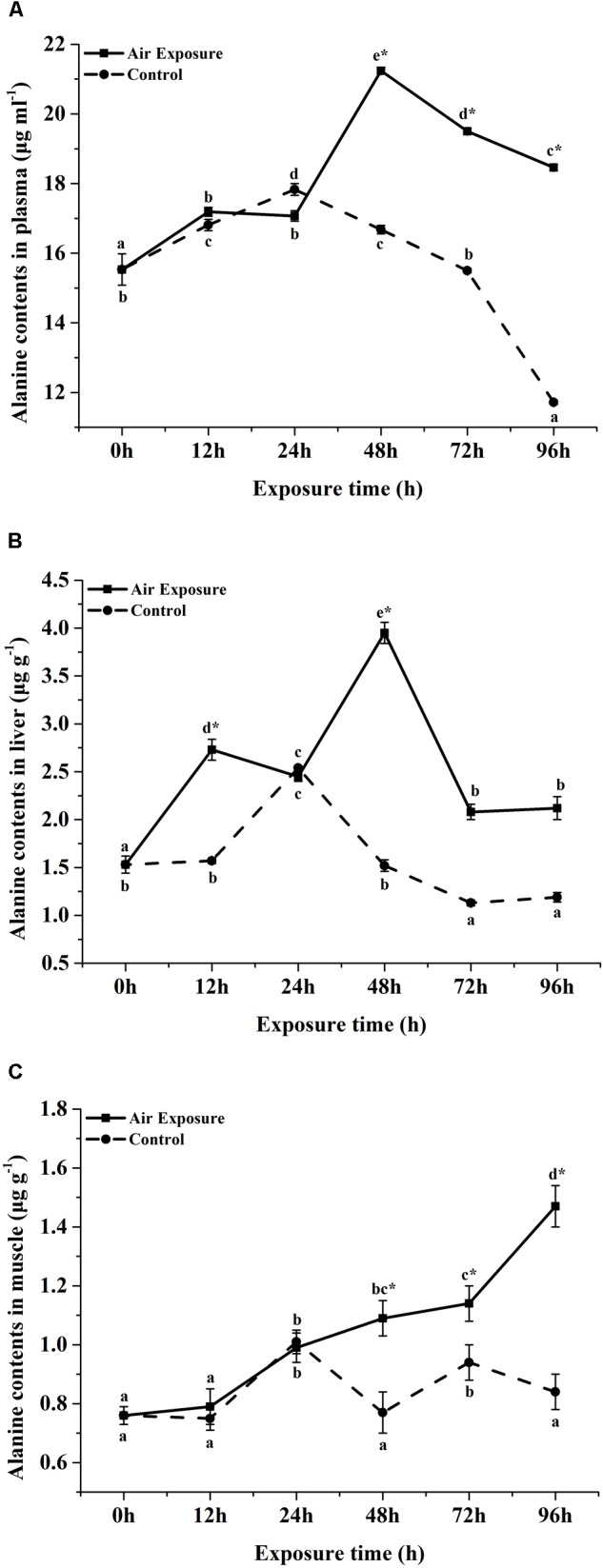
Effects of various periods of air exposure on the contents of the affected FAA in the plasma **(A)**, liver **(B)** and muscle **(C)** of *P. dabryanus.* The different lowercase letter indicate significant difference among different exposure times in control group, the capital letters indicate significant difference among different exposure times in air exposure group, ^∗^indicate significant difference between air exposure group and control group.

FAA contents in plasma of *P. dabryanus* during different periods of aerial exposure were summarized in Table 1. Most of the detected FAA concentrations were significantly affected by the various periods of aerial exposure (*P* < 0.05). At the same sampling time, the contents of plasma free threonine, serine, glutamate, glycine, methionine, tyrosine, phenylalanine, histidine, and arginine were significantly lower than these values in the control group (*P* < 0.05). After 12 h of aerial exposure, the plasma glutamate contents increased significantly (*P* < 0.05) and reached peak value at 24 h of air exposure. Then the values decreased remarkably until the end of the experiment (*P* < 0.05). At 24, 48, and 96 h of aerial exposure, the plasma glutamate concentrations were markedly lower than these values in the control group (*P* < 0.05). With the increasing amount of air exposure time, the plasma alanine contents increased significantly and reached dramatically higher levels than the control values (*P* < 0.05, Figure 1A).

**Table 1:** Effects of various periods of aerial exposure on the contents (μg ml^-1^, *n* = 3) of the affected FAA in the plasma of *Paramisgurnus dabryanus.*

FAA	Group	0 h	12 h	24 h	48 h	72 h	96 h
P-Ser	C	nd	1.74 ± 0.04	2.06 ± 0.03	2.08 ± 0.01	1.66 ± 0.03	2.25 ± 0.04
	T	nd	nd	4.34 ± 0.06	6.00 ± 0.13	2.62 ± 0.04	2.98 ± 0.04
Tau	C	15.63 ± 0.38^d^	14.77 ± 0.08^c^	13.15 ± 0.05^b^	25.55 ± 0.06^e^	10.07 ± 0.05^a^	12.97 ± 0.19^b^
	T	15.63 ± 0.38^b^	22.26 ± 0.18^c^*	11.66 ± 0.20^a^*	23.68 ± 0.78^d^	26.14 ± 0.09^e^*	11.23 ± 0.10^a^*
Thr	C	36.97 ± 0.82^b^	33.26 ± 0.14^a^	71.64 ± 0.15^e^	88.75 ± 0.23^f^	43.81 ± 0.20^c^	56.02 ± 0.21^d^
	T	36.97 ± 0.82^d^	31.66 ± 0.24^b^*	35.16 ± 0.77^c^*	35.75 ± 0.45^c^*	30.64 ± 0.10^a^*	29.97 ± 0.16^a^*
Ser	C	16.71 ± 0.39^e^	13.18 ± 0.12^b^	23.53 ± 0.13^f^	15.59 ± 0.10^d^	11.41 ± 0.11^a^	15.35 ± 0.31^c^
	T	16.71 ± 0.39^f^	10.84 ± 0.24^d^*	10.20 ± 0.17^c^*	8.95 ± 0.68^b^*	12.18 ± 0.15^e^*	8.21 ± 0.09^a^*
Glu	C	47.76 ± 0.58^c^	38.15 ± 0.14^a^	66.07 ± 0.25^f^	53.47 ± 0.31^e^	43.27 ± 0.15^b^	52.14 ± 0.36^d^
	T	47.76 ± 0.58^e^	39.13 ± 0.47^b^*	58.72 ± 0.65^f^*	36.52 ± 0.34^a^*	45.94 ± 0.14^d^*	45.15 ± 0.12^c^*
Gly	C	14.01 ± 0.21^d^	13.74 ± 0.08^c^	17.14 ± 0.11^f^	13.05 ± 0.05^b^	10.63 ± 0.12^a^	15.19 ± 0.07^e^
	T	14.01 ± 0.21^d^	14.32 ± 0.07^e^*	13.58 ± 0.14^c^*	12.53 ± 0.11^a^*	13.04 ± 0.08^b^*	14.60 ± 0.13^f^*
Ala	C	15.53 ± 0.45^b^	16.81 ± 0.16^c^	17.83 ± 0.17^d^	16.68 ± 0.12^c^	15.50 ± 0.06^b^	11.72 ± 0.06^a^
	T	15.53 ± 0.45^a^	17.19 ± 0.13^b^*	17.07 ± 0.15^b^*	21.24 ± 0.10^e^*	19.50 ± 0.08^d^*	18.46 ± 0.08^c^*
α-ABA	C	1.16 ± 0.05^b^	0.56 ± 0.05^a^	2.72 ± 0.07^e^	1.78 ± 0.03^d^	1.18 ± 0.08^b^	1.37 ± 0.03^c^
	T	1.16 ± 0.05^b^	1.14 ± 0.04^b^*	1.68 ± 0.06^c^*	1.00 ± 0.06^a^*	2.40 ± 0.06^d^*	1.68 ± 0.03^c^*
Val	C	54.26 ± 0.38^e^	29.84 ± 0.20^a^	51.03 ± 0.46^b^	53.56 ± 0.13^d^	52.35 ± 0.21^c^	54.71 ± 0.46^e^
	T	54.26 ± 0.38^b^	44.84 ± 0.14^a^*	59.79 ± 0.36^c^*	60.21 ± 0.14^c^*	81.78 ± 0.26^d^*	84.89 ± 0.12^e^*
Cys	C	2.30 ± 0.10	2.84 ± 0.07	2.42 ± 0.09	2.42 ± 0.07	nd	1.97 ± 0.01
	T	2.30 ± 0.10	2.43 ± 0.08	2.04 ± 0.08	0.43 ± 0.03	0.91 ± 0.06	nd
Met	C	15.74 ± 0.30^d^	12.80 ± 0.06^b^	14.65 ± 0.13^c^	16.05 ± 0.05^e^	10.77 ± 0.08^a^	15.58 ± 0.08^d^
	T	15.74 ± 0.30^e^	14.33 ± 0.11^d^*	13.88 ± 0.32^c^*	8.28 ± 0.11^b^*	7.56 ± 0.09^a^*	7.67 ± 0.06^a^*
Ile	C	35.19 ± 0.21^d^	17.14 ± 0.05^a^	33.67 ± 0.22^c^	32.45 ± 0.06^b^	39.60 ± 0.21^f^	36.87 ± 0.19^e^
	T	35.19 ± 0.21^b^	27.82 ± 0.16^a^*	39.98 ± 0.32^c^*	45.09 ± 0.45^d^*	64.95 ± 0.16^e^*	68.92 ± 0.22^f^*
Leu	C	66.16 ± 0.27^d^	34.75 ± 0.13^a^	59.86 ± 0.28^b^	60.83 ± 0.12^c^	71.42 ± 0.42^f^	68.76 ± 0.30^e^
	T	66.16 ± 0.27^b^	53.41 ± 0.23^a^*	77.76 ± 0.36^c^*	80.34 ± 0.19^d^*	110.60 ± 0.56^e^*	122.61 ± 0.22^f^*
Tyr	C	16.23 ± 0.13^f^	10.10 ± 0.10^b^	11.09 ± 0.09^c^	8.78 ± 0.08^a^	11.88 ± 0.08^d^	16.38 ± 0.06^f^
	T	16.23 ± 0.13^f^	10.19 ± 0.12^b^	8.04 ± 0.09^a^*	11.86 ± 0.10^e^*	10.47 ± 0.13^c^*	10.83 ± 0.12^d^*
Phe	C	20.46 ± 0.28^f^	12.41 ± 0.11^c^	10.84 ± 0.04^a^	13.42 ± 0.21^d^	11.65 ± 0.05^b^	13.77 ± 0.04^e^
	T	20.46 ± 0.28^e^	7.34 ± 0.11^a^*	8.41 ± 0.24^b^*	8.46 ± 0.10^b^*	9.24 ± 0.12^c^*	11.76 ± 0.07^d^*
Orn	C	3.14 ± 0.06^a^	3.81 ± 0.07^b^	6.75 ± 0.11^e^	4.99 ± 0.04^c^	3.89 ± 0.02^b^	5.78 ± 0.08^d^
	T	3.14 ± 0.06^a^	5.06 ± 0.06^b^*	9.28 ± 0.07^e^*	6.86 ± 0.11^d^*	5.44 ± 0.20^c^*	5.37 ± 0.06^c^*
His	C	22.99 ± 0.18^e^	21.91 ± 0.08^d^	21.79 ± 0.14^d^	18.06 ± 0.11^a^	16.46 ± 0.06^c^	16.12 ± 0.11^b^
	T	22.99 ± 0.18^f^	19.77 ± 0.11^e^*	17.81 ± 0.24^d^*	8.42 ± 0.11^a^*	12.32 ± 0.10^b^*	12.77 ± 0.10^c^*
Arg	C	20.30 ± 0.22^c^	15.58 ± 0.18^a^	23.72 ± 0.22^d^	25.12 ± 0.10^e^	18.63 ± 0.09^b^	26.81 ± 0.06^f^
	T	20.30 ± 0.22^a^	20.74 ± 0.19^b^*	27.46 ± 0.32^e^*	28.73 ± 0.08^f^*	21.95 ± 0.08^c^*	26.93 ± 0.29^d^*
Hypro	C	nd	5.22 ± 0.12	nd	1.92 ± 0.02	nd	2.63 ± 0.02
	T	nd	nd	nd	nd	nd	nd
Pro	C	7.03 ± 0.17	5.71 ± 0.06	7.65 ± 0.04	nd	nd	5.88 ± 0.08
	T	7.03 ± 0.17	2.08 ± 0.09	7.64 ± 0.21	5.41 ± 0.09	7.74 ± 0.21	6.05 ± 0.05

*C is control group, T is air exposure group. The different upperscripts in same line are significant difference among the different exposure time. ^∗^ is significant difference between air exposure group and control group. P-Ser; Phosphoserine, Tau; Taurine, Thr; Threonine, Ser; Serine, Glu; Glutamate, Gly; Glycine, Ala; Alanin, α-ABA; α-Amino-n-butyric Acid, Val; Valine, Cys; Cystine, Met; Methionine, Ile; Isoleucine, Leu; Leucine, Tyr; Tyrosine, Phe; Phenylalanine, Orn; Ornithine, His; Histidine, Arg; Arginine, Hypro; Hydroxy-L-proline, Pro; Proline.*

### Liver FAA Contents

The liver FAA contents of *P. dabryanus* during different periods of aerial exposure were shown in Table 2. Most of detected liver FAA concentrations were significantly affected by the various periods of aerial exposure (*P* < 0.05). From 24 to 48 h of aerial exposure, the liver free glutamate contents increased significantly and reached a peak value at 48 h of air exposure (*P* < 0.05). Thereafter, this content rapidly decreased to a constant value until the end of the experiment. Meanwhile, all the liver free glutamate contents in aerial exposure group were remarkably lower than these values in the control group (*P* < 0.05). The liver free alanine concentrations increased dramatically during the first 24 h of freshwater exposure and 48 h of air exposure (*P* < 0.05, Figure 1B), and then, a significant decrease was observed in both the control and aerial exposure groups (*P* < 0.05, Figure 1B). Furthermore, the free alanine contents in the air exposure group were markedly higher than equivalent values in control group (*P* < 0.05, Figure 1B). With the increase in air exposure time, the liver free arginine and proline contents firstly rised and later decreased, and all the experimental values in air exposure group were remarkably higher than such values in control group (*P* < 0.05).

**Table 2:** Effects of various periods of aerial exposure on the contents (mg g^-1^ dry weight, *n* = 3) of the affected FAA in the liver of *P. dabryanus.*

FAA	Group	0 h	12 h	24 h	48 h	72 h	96 h
P-Ser	C	1.38 ± 0.04^a^	1.57 ± 0.06^bc^	1.37 ± 0.04^a^	1.41 ± 0.05^a^	1.47 ± 0.07^ab^	1.66 ± 0.11^d^
	T	1.38 ± 0.04^d^	1.58 ± 0.08^e^	1.41 ± 0.09^d^	1.23 ± 0.08^c∗^	0.85 ± 0.03^a∗^	1.01 ± 0.06^b∗^
Tau	C	2.01 ± 0.08^b^	1.79 ± 0.09^a^	2.69 ± 0.12^c^	2.13 ± 0.11^b^	2.11 ± 0.09^b^	3.79 ± 0.19^d^
	T	2.01 ± 0.08^a^	3.50 ± 0.12^c∗^	3.91 ± 0.28^d∗^	4.94 ± 0.19^e∗^	2.99 ± 0.09^b∗^	3.17 ± 0.07^b∗^
PEA	C	1.80 ± 0.07	2.00 ± 0.04	1.94 ± 0.06	1.98 ± 0.07	1.59 ± 0.07	2.49 ± 0.17
	T	1.80 ± 0.07	nd	nd	nd	nd	nd
Asp	C	0.68 ± 0.03^bc^	1.23 ± 0.03^d^	1.18 ± 0.07^d^	0.73 ± 0.03^c^	0.50 ± 0.04^a^	0.61 ± 0.06^b^
	T	0.68 ± 0.03^a^	1.53 ± 0.03^e∗^	1.31 ± 0.07^d^	2.05 ± 0.07^f∗^	0.97 ± 0.04^b∗^	1.07 ± 0.05^c∗^
Thr	C	0.70 ± 0.06^b^	0.68 ± 0.02^b^	1.19 ± 0.05^d^	0.81 ± 0.02^c^	0.57 ± 0.05^a^	0.76 ± 0.08^bc^
	T	0.70 ± 0.06^a^	1.45 ± 0.05^c∗^	1.43 ± 0.08^c∗^	2.55 ± 0.07^d∗^	1.15 ± 0.05^b∗^	1.14 ± 0.04^b∗^
Ser	C	0.57 ± 0.08^c^	0.56 ± 0.04^c^	0.86 ± 0.06^d^	0.47 ± 0.04^b^	0.32 ± 0.02^a^	0.44 ± 0.03^b^
	T	0.57 ± 0.08^a^	1.21 ± 0.07^d∗^	1.05 ± 0.02^c∗^	1.75 ± 0.04^e∗^	0.89 ± 0.06^b∗^	0.85 ± 0.02^b∗^
Glu	C	0.33 ± 0.02^a^	1.80 ± 0.05^c^	2.58 ± 0.13^e^	1.88 ± 0.08^c^	1.42 ± 0.06^b^	2.18 ± 0.15^d^
	T	0.33 ± 0.02^b^	0.37 ± 0.06^b∗^	0.31 ± 0.01^b∗^	0.49 ± 0.05^c∗^	0.19 ± 0.02^a∗^	0.21 ± 0.02^a∗^
Gly	C	0.31 ± 0.02^b^	0.31 ± 0.01^b^	0.51 ± 0.01^c^	0.30 ± 0.02^b^	0.24 ± 0.01^a^	0.79 ± 0.04^d^
	T	0.31 ± 0.02^a^	0.57 ± 0.06^b∗^	0.60 ± 0.08^b^	0.94 ± 0.02^c∗^	0.56 ± 0.04^b∗^	0.55 ± 0.04^b∗^
Ala	C	1.53 ± 0.09^b^	1.57 ± 0.03^b^	2.54 ± 0.02^c^	1.52 ± 0.06^b^	1.13 ± 0.03^a^	1.19 ± 0.05^a^
	T	1.53 ± 0.09^a^	2.73 ± 0.11^d∗^	2.45 ± 0.05^c^	3.95 ± 0.11^e∗^	2.08 ± 0.08^b∗^	2.12 ± 0.12^b∗^
α-ABA	C	0.06 ± 0.02	nd	nd	nd	nd	0.09 ± 0.01
	T	0.06 ± 0.02	0.05 ± 0.01	0.08 ± 0.01	0.10 ± 0.01	0.06 ± 0.01	0.07 ± 0.01
Val	C	0.81 ± 0.09^c^	0.76 ± 0.04^bc^	1.18 ± 0.08^e^	0.66 ± 0.03^b^	0.52 ± 0.02^a^	1.05 ± 0.09^d^
	T	0.81 ± 0.09^a^	1.92 ± 0.03^c∗^	1.56 ± 0.06^b∗^	3.18 ± 0.08^d∗^	1.46 ± 0.06^b∗^	1.54 ± 0.04^b∗^
Met	C	0.34 ± 0.04^d^	0.31 ± 0.01^cd^	0.43 ± 0.03^e^	0.21 ± 0.00^b^	0.15 ± 0.01^a^	0.29 ± 0.03^c^
	T	0.34 ± 0.04^a^	0.75 ± 0.05^c∗^	0.71 ± 0.04^c∗^	1.39 ± 0.09^d∗^	0.53 ± 0.03^b∗^	0.59 ± 0.03^b∗^
Cysthi	C	0.26 ± 0.05^d^	0.21 ± 0.00^bc^	0.18 ± 0.00^b^	0.17 ± 0.01^ab^	0.13 ± 0.00^a^	0.24 ± 0.03^cd^
	T	0.26 ± 0.05^c^	0.19 ± 0.02^b^	0.25 ± 0.03^c^	0.24 ± 0.02^bc^	0.12 ± 0.01^a^	0.19 ± 0.02^b^
Ile	C	0.62 ± 0.02^c^	0.58 ± 0.03^c^	0.83 ± 0.03^d^	0.48 ± 0.04^b^	0.38 ± 0.02^a^	0.35 ± 0.04^a^
	T	0.62 ± 0.02^a^	1.49 ± 0.06^d∗^	1.21 ± 0.06^c∗^	2.48 ± 0.06^e∗^	1.08 ± 0.07^b∗^	1.18 ± 0.08^bc∗^
Leu	C	1.24 ± 0.13^d^	1.10 ± 0.07^c^	1.76 ± 0.06^e^	0.91 ± 0.01^b^	0.67 ± 0.05^a^	1.25 ± 0.06^d^
	T	1.24 ± 0.13^a^	3.09 ± 0.12^c∗^	2.48 ± 0.08^b∗^	5.34 ± 0.13^d∗^	2.31 ± 0.09^b∗^	2.40 ± 0.07^b∗^
Tyr	C	0.73 ± 0.04^d^	0.63 ± 0.03^c^	0.91 ± 0.08^e^	0.48 ± 0.05^b^	0.35 ± 0.03^a^	0.87 ± 0.05^e^
	T	0.73 ± 0.04^a^	1.83 ± 0.06^d∗^	1.38 ± 0.05^c∗^	3.10 ± 0.10^e∗^	1.17 ± 0.03^b∗^	1.40 ± 0.05^c∗^
Phe	C	0.74 ± 0.08^c^	0.49 ± 0.02^b^	1.12 ± 0.07^d^	0.49 ± 0.07^b^	0.20 ± 0.01^a^	0.81 ± 0.06^c^
	T	0.74 ± 0.08^a^	2.15 ± 0.05^d∗^	1.71 ± 0.09^c∗^	3.54 ± 0.08^e∗^	1.35 ± 0.05^b∗^	1.61 ± 0.04^c∗^
β-Ala	C	0.43 ± 0.03^bc^	0.41 ± 0.01^b^	0.70 ± 0.04^d^	0.38 ± 0.02^b^	0.29 ± 0.04^a^	0.47 ± 0.04^c^
	T	0.43 ± 0.03^a^	1.29 ± 0.07^e∗^	1.00 ± 0.04^c∗^	1.84 ± 0.04^f∗^	0.79 ± 0.01^b∗^	1.04 ± 0.02^d∗^
β-AiBA	C	0.15 ± 0.02^c^	0.12 ± 0.01^ab^	0.27 ± 0.02^d^	0.14 ± 0.01^bc^	0.09 ± 0.01^a^	0.72 ± 0.02^e^
	T	0.15 ± 0.02^a^	0.73 ± 0.03^d∗^	0.65 ± 0.05^c∗^	1.26 ± 0.06^e∗^	0.42 ± 0.02^b∗^	0.64 ± 0.04^c∗^
γ-ABA	C	0.17 ± 0.07^cd^	0.11 ± 0.00^ab^	0.10 ± 0.00^a^	0.16 ± 0.00^bc^	0.07 ± 0.00^a^	0.22 ± 0.01^d^
	T	0.17 ± 0.07^a^	0.34 ± 0.02^b∗^	0.34 ± 0.02^b∗^	0.59 ± 0.05^c∗^	0.18 ± 0.02^a∗^	0.33 ± 0.02^b∗^
Trp	C	0.27 ± 0.08	nd	0.33 ± 0.03	0.16 ± 0.01	nd	0.12 ± 0.02
	T	0.27 ± 0.08	1.06 ± 0.04	0.97 ± 0.04	2.09 ± 0.07	0.50 ± 0.01	0.99 ± 0.03
Orn	C	0.05 ± 0.02	nd	nd	nd	nd	0.17 ± 0.01
	T	0.05 ± 0.02	0.12 ± 0.01	0.13 ± 0.02	0.27 ± 0.02	0.15 ± 0.00	0.22 ± 0.01
His	C	0.22 ± 0.02	0.18 ± 0.01	0.23 ± 0.02	0.11 ± 0.01	nd	0.24 ± 0.03
	T	0.22 ± 0.02	0.45 ± 0.04	0.38 ± 0.03	0.74 ± 0.04	0.30 ± 0.02	0.30 ± 0.02
Arg	C	1.71 ± 0.10^d^	1.30 ± 0.06^c^	2.02 ± 0.13^e^	0.90 ± 0.06^b^	0.57 ± 0.04^a^	0.66 ± 0.06^a^
	T	1.71 ± 0.10^a^	4.05 ± 0.05^e∗^	2.76 ± 0.06^d∗^	6.44 ± 0.14^f∗^	2.28 ± 0.08^b∗^	2.49 ± 0.09^c∗^
Pro	C	0.53 ± 0.03^d^	0.27 ± 0.02^a^	0.59 ± 0.03^e^	0.41 ± 0.04^c^	0.27 ± 0.02^a^	0.32 ± 0.03^b^
	T	0.53 ± 0.03^a^	0.74 ± 0.03^c∗^	0.73 ± 0.02^c∗^	1.11 ± 0.08^d∗^	0.61 ± 0.03^b∗^	0.59 ± 0.03^ab∗^

*C is control group, T is air exposure group. The different upperscripts in same line are significant difference among the different exposure time. ^∗^ is significant difference between air exposure group and control group, nd is not detected. PEA; Phosphoethanolamine, Asp; Aspartic Acid, Cysthi; Cystathionine, β-Ala; β-Alanine, β-AiBA; β-Aminoisobutyric Acid, γ-ABA; γ-Aminobutyric Acid, Trp; Tryptophan.*

### Muscle FAA Contents

Similarly, most of detected muscle FAA concentrations were significantly affected by the various periods of aerial exposure (Table 3, *P* < 0.05). After 72 h of air exposure, the muscle free glutamate contents increased markedly (*P* < 0.05) and were significantly higher than the control values (*P* < 0.05). The muscle free alanine contents remained at constant values during the first 12 h of aerial exposure (*P* > 0.05, Figure 1C), thereafter, this concentrations increased significantly till the end of experiment (*P* < 0.05, Figure 1C). After 48 h of air exposure, the muscle free alanine concentrations were markedly higher than this values in the control group (*P* < 0.05, Figure 1C). The muscle free arginine and proline contents increased remarkably after 72 h of aerial exposure (*P* < 0.05) and became significantly higher than the control values (*P* < 0.05).

**Table 3:** Effects of various periods of aerial exposure on the contents (mg g^-1^ dry weight, *n* = 3) of the affected FAA in the muscle of *P. dabryanus.*

FAA	Group	0 h	12 h	24 h	48 h	72 h	96 h
P-Ser	C	0.03 ± 0.00^a^	0.04 ± 0.00^b^	0.09 ± 0.01^f^	0.05 ± 0.00^c^	0.06 ± 0.00^d^	0.07 ± 0.01^e^
	T	0.03 ± 0.00^a^	0.05 ± 0.01^b^	0.07 ± 0.01^c^	0.05 ± 0.01^b^	0.05 ± 0.01^b^	0.07 ± 0.01^c^
Tau	C	1.07 ± 0.04^b^	1.07 ± 0.06^b^	1.08 ± 0.06^b^	0.95 ± 0.05^a^	1.55 ± 0.11^d^	1.40 ± 0.10^b^
	T	1.07 ± 0.04^b^	1.02 ± 0.06^ab^	0.92 ± 0.08^a^	1.46 ± 0.07^c∗^	1.61 ± 0.09^d^	1.89 ± 0.09^e∗^
Asp	C	0.12 ± 0.02^ab^	0.12 ± 0.01^ab^	0.15 ± 0.03^c^	0.13 ± 0.02^c^	0.14 ± 0.01^c^	0.09 ± 0.01^a^
	T	0.12 ± 0.02^bc^	0.10 ± 0.01^b^	0.13 ± 0.01^d^	0.08 ± 0.01^a∗^	0.12 ± 0.01^bc^	0.08 ± 0.01^a^
Thr	C	0.66 ± 0.05^a^	0.74 ± 0.04^a^	0.90 ± 0.07^b^	0.95 ± 0.07^b^	0.98 ± 0.08^b^	0.99 ± 0.07^b^
	T	0.66 ± 0.05^a^	0.91 ± 0.07^b∗^	1.14 ± 0.09^c∗^	1.05 ± 0.04^c^	1.03 ± 0.04^c^	1.35 ± 0.08^d∗^
Ser	C	0.54 ± 0.03^b^	0.45 ± 0.05^a^	0.74 ± 0.04^d^	0.62 ± 0.03^c^	0.57 ± 0.05^bc^	0.64 ± 0.05^c^
	T	0.54 ± 0.03^a^	0.68 ± 0.05^b∗^	0.70 ± 0.04^bc^	0.78 ± 0.06^cd∗^	0.80 ± 0.05^de∗^	0.89 ± 0.07^e∗^
Glu	C	0.14 ± 0.02^a^	0.23 ± 0.03^cd^	0.15 ± 0.02^ab^	0.19 ± 0.02^bc^	0.23 ± 0.03^cd^	0.27 ± 0.03^d^
	T	0.14 ± 0.02^a^	0.15 ± 0.03^a∗^	0.17 ± 0.02^a^	0.23 ± 0.03^b^	0.30 ± 0.02^c∗^	0.36 ± 0.03^d∗^
Gly	C	0.86 ± 0.04^a^	0.92 ± 0.08^a^	1.52 ± 0.06^c^	1.18 ± 0.06^b^	1.19 ± 0.08^b^	1.59 ± 0.09^c^
	T	0.86 ± 0.04^a^	1.52 ± 0.08^b∗^	1.58 ± 0.08^b^	1.58 ± 0.08^b∗^	1.52 ± 0.11^b∗^	2.23 ± 0.13^c∗^
Ala	C	0.76 ± 0.03^a^	0.75 ± 0.04^a^	1.01 ± 0.04^b^	0.77 ± 0.04^a^	0.94 ± 0.06^b^	0.84 ± 0.06^a^
	T	0.76 ± 0.03^a^	0.79 ± 0.06^a^	0.99 ± 0.05^b^	1.09 ± 0.06^bc∗^	1.14 ± 0.06^c∗^	1.47 ± 0.07^d∗^
Val	C	0.20 ± 0.01^b^	0.15 ± 0.02^a^	0.24 ± 0.02^bc^	0.22 ± 0.02^b^	0.33 ± 0.03^d^	0.28 ± 0.03^c^
	T	0.20 ± 0.01^a^	0.19 ± 0.02^a^	0.30 ± 0.03^b∗^	0.33 ± 0.04^b∗^	0.40 ± 0.03^c∗^	0.52 ± 0.02^d∗^
Met	C	0.10 ± 0.01^b^	0.09 ± 0.01^a^	0.12 ± 0.01^c^	0.09 ± 0.00^ab^	0.10 ± 0.01^b^	0.14 ± 0.01^d^
	T	0.10 ± 0.01^ab^	0.10 ± 0.01^ab^	0.12 ± 0.01^bc^	0.10 ± 0.02^a^	0.10 ± 0.01^ab^	0.13 ± 0.01^c^
Ile	C	0.15 ± 0.01^b^	0.10 ± 0.01^a^	0.17 ± 0.03^bc^	0.16 ± 0.03^b^	0.27 ± 0.04^d^	0.21 ± 0.02^c^
	T	0.15 ± 0.01^a^	0.12 ± 0.02^a^	0.21 ± 0.02^b^	0.26 ± 0.06^b^	0.32 ± 0.02^c^	0.42 ± 0.04^d∗^
Leu	C	0.29 ± 0.03^b^	0.22 ± 0.03^a^	0.30 ± 0.05^b^	0.31 ± 0.04^b^	0.51 ± 0.03^d^	0.40 ± 0.04^c^
	T	0.29 ± 0.03^a^	0.25 ± 0.03^a^	0.40 ± 0.05^b^	0.46 ± 0.05^b∗^	0.57 ± 0.06^c^	0.74 ± 0.05^d∗^
Tyr	C	0.11 ± 0.01^c^	0.06 ± 0.00^a^	0.11 ± 0.01^c^	0.08 ± 0.01^b^	0.09 ± 0.00^b^	0.12 ± 0.02^c^
	T	0.11 ± 0.01^a^	0.09 ± 0.01^a∗^	0.10 ± 0.01^a^	0.11 ± 0.01^a∗^	0.10 ± 0.01^a^	0.13 ± 0.01^b^
Phe	C	0.12 ± 0.00^b^	0.07 ± 0.00^a^	0.12 ± 0.02^b^	0.08 ± 0.01^a^	0.12 ± 0.01^b^	0.12 ± 0.01^b^
	T	0.12 ± 0.00^b^	0.07 ± 0.00^a^	0.11 ± 0.02^b^	0.10 ± 0.01^b∗^	0.11 ± 0.01^b^	0.17 ± 0.02^c∗^
Trp	C	0.08 ± 0.00^c^	0.05 ± 0.00^a^	0.10 ± 0.02^d^	0.06 ± 0.00^ab^	0.07 ± 0.01^bc^	0.08 ± 0.00^c^
	T	0.08 ± 0.00^ab^	0.06 ± 0.01^a^	0.08 ± 0.01^ab^	0.09 ± 0.01^b∗^	0.09 ± 0.00^b∗^	0.13 ± 0.03^c∗^
Orn	C	0.03 ± 0.00^a^	0.03 ± 0.00^a^	0.04 ± 0.00^b^	0.04 ± 0.01^b^	0.04 ± 0.00^b^	0.04 ± 0.00^b^
	T	0.03 ± 0.00^a^	0.04 ± 0.00^ab^	0.04 ± 0.01^b^	0.05 ± 0.01^c^	0.05 ± 0.01^c^	0.06 ± 0.01^d∗^
His	C	1.68 ± 0.08^a^	1.80 ± 0.09^ab^	2.30 ± 0.15^c^	1.60 ± 0.08^a^	1.95 ± 0.15^b^	2.26 ± 0.17^c^
	T	1.68 ± 0.08^a^	2.25 ± 0.12^b∗^	2.47 ± 0.18^bc^	2.58 ± 0.14^c∗^	2.67 ± 0.08^c∗^	4.28 ± 0.26^d∗^
3-Mehis	C	0.07 ± 0.01^c^	0.06 ± 0.01^bc^	0.05 ± 0.00^a^	0.05 ± 0.00^a^	0.06 ± 0.00^b^	0.05 ± 0.00^a^
	T	0.07 ± 0.01^b^	0.05 ± 0.01^a^	0.04 ± 0.01^a^	0.04 ± 0.01^a^	0.04 ± 0.00^a∗^	0.08 ± 0.01^b∗^
Arg	C	0.23 ± 0.03^bcd^	0.16 ± 0.03^a^	0.29 ± 0.03^d^	0.19 ± 0.03^ab^	0.22 ± 0.02^abc^	0.28 ± 0.05^cd^
	T	0.23 ± 0.03^a^	0.24 ± 0.02^a∗^	0.26 ± 0.03^a^	0.24 ± 0.01^a^	0.29 ± 0.04^a^	0.47 ± 0.06^b∗^
Hypro	C	0.07 ± 0.01^a^	0.14 ± 0.02^c^	0.10 ± 0.00^b^	0.10 ± 0.01^b^	0.08 ± 0.01^ab^	0.09 ± 0.01^ab^
	T	0.07 ± 0.01^a^	0.09 ± 0.01^b∗^	0.09 ± 0.01^b^	0.10 ± 0.01^b^	0.07 ± 0.01^a^	0.10 ± 0.01^b^
Pro	C	0.29 ± 0.06^a^	0.39 ± 0.06^bc^	0.37 ± 0.04^ab^	0.51 ± 0.05^d^	0.46 ± 0.04^cd^	0.32 ± 0.02^ab^
	T	0.29 ± 0.06^a^	0.38 ± 0.06^b^	0.65 ± 0.07^c∗^	0.38 ± 0.04^b∗^	0.39 ± 0.05^b^	0.59 ± 0.04^c∗^

*C is control group, T is air exposure group. The different upperscripts in same line are significant difference among the different exposure time. ^∗^ is significant difference between air exposure group and control group, nd is not detected. 3-Mehis; 3-Methylhistidin.*

## Discussion

In teleost, reduction of proteolysis and amino acids metabolism should be an effective strategy for ammonia detoxification, by reduction of endogenous ammonia production (Ip and Chew, 2010; Chew and Ip, 2014). However, amino acid catabolism as the energy source was also inhibited in this situation. Apparently, it was inapplicable for several species which required a large quantity of energy to deal with ammonia loading, such as *P. schlosseri* (Kok et al., 1998). To overcome this, some air-breathing fishes adopted the strategies of partial amino acid catabolism in conjunction with the reduction of endogenous ammonia production (Chew and Ip, 2014). A lot of certain amino acids can be partially metabolized without internal ammonia production, forming alanine (Tsui et al., 2004). For instance, 1 mol of glutamate can be catabolized to alanine, yielding 10 mol of ATP (Tsui et al., 2004).

**FIGURE 2:**
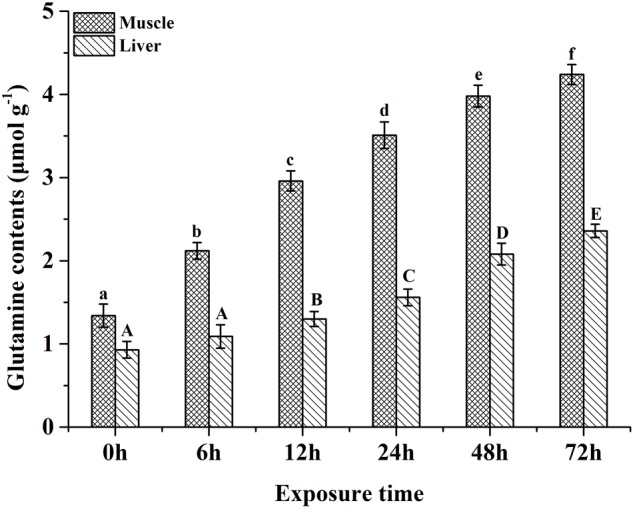
Effects of various periods of air exposure on the contents of the affected glutamine in the muscle and liver of *P. dabryanus* (Zhang et al., 2017a). The different superscripts in same tissue are statistical difference (*P <* 0.05).

Glutamine synthesis has been proven to play an important role in ammonia tolerance in many fish species, such as *Oxyeleotris marnorata* (Jow et al., 1999), *M. anguillicaudatus* (Chew et al., 2001), *Bostrichyths sinensis* (Ip et al., 2001a), *M. albus* (Tay et al., 2003), *Oncorhynchus mykiss* (Sanderson et al., 2010) and *Apostichopus japonicus* (Wang et al., 2014). In fish species, glutamate and NH_4_^+^ can be synthesized to glutamine by glutamine synthetase. In the present study, the plasma, liver and muscle free glutamate of *P. dabryanus* during aerial exposure were significantly lower than these values in control group. Furthermore, our previous study reported that the tissue glutamine contents and glutamine synthetase activities increased markedly as air exposure time increased (Figure 2) (Zhang et al., 2017a). Therefore, our results showed that glutamate and NH_4_^+^ are used to synthesize glutamine via glutamine synthetase to convert internal ammonia into non-toxic glutamine in *P. dabryanus* during air exposure. The synthesized glutamine can be stored in the organism for succedent usage in various metabolic procedures (e.g., syntheses of purines, pyrimidines and mucopolysaccharides) when favorable surrounding conditions recur (Chew and Ip, 2014). The previous studies have also demonstrated that glutamine synthesis was an effective ammonia detoxification strategy in several fish species, including *Clarias gariepinus* (Wee et al., 2007), *M. albus* (Tng et al., 2009) and *O. mykiss* (Sanderson et al., 2010).

Many amino acids can be partially metabolized to alanine without releasing ammonia via transamination, such as glutamate, arginine and proline (Tsui et al., 2004; Ip and Chew, 2010; Chew and Ip, 2014; Zhang et al., 2017c). From this perspective, partial amino acid metabolism leading to alanine form may be an effective ammonia defensive strategy or at least a process to suppress the production of endogenous ammonia (Zhang et al., 2017b). In the present study, obvious increases inplasma, liver and muscle free alanine contents in *P. dabryanus* were observed during different periods of aerial exposure. Similar results were also found in *M. anguillicaudatus* (Chew et al., 2001), *P. schlosseri* (Ip et al., 2001b) and *C. asiatica* (Chew et al., 2003b). Our results suggested that the *P. dabryanus* could catabolize several certain amino acids, leading alanine form to reduce endogenous ammonia production. The decrease in tissue free glutamate, arginine and proline in *P. dabryanus* indicated that these certain amino acids should be the starting substrate to be converted to alanine and energy. Furthermore, the plasma glucose and lactate contents in *P. dabryanus* increased significantly during various periods of air exposure (unpublished data). Taken together, our results revealed that the start of the ammonia detoxification strategy of partial amino acid catabolism was related to the body energy expenditure and air exposure time.

## Conclusion

Overall, most of detected plasma, liver and muscle FAA concentrations in *P. dabryanus* were significantly affected by the various periods of aerial exposure time. The plasma, liver and muscle free glutamate of *P. dabryanus* were significantly lower than these values in the control group during aerial exposure. Furthermore, the tissue glutamine contents and glutamine synthetase activities increased markedly with the increase in air exposure time. Our results showed that glutamate and NH_4_^+^ would be used to synthesize glutamine via glutamine synthetase to convert internal ammonia into non-toxic glutamine in *P. dabryanus* during air exposure. Obvious increases in plasma, liver and muscle free alanine contents in *P. dabryanus* were observed during different periods of aerial exposure, suggesting that the *P. dabryanus* could catabolize several certain amino acids, leading alanine form to reduce endogenous ammonia production. The decrease in tissue free glutamate, arginine and proline in *P. dabryanus* indicated that these certain amino acids should be the starting substrate to be converted to alanine and energy.

## Author Contributions

Y-LZ conceived the ideas and designed the experiments, performed all the experiments, conducted statistical analysis, prepared the tables, and wrote the manuscript. G-YW, Z-HZ, Y-YX, HJ, and Z-RD helped in sample collection and with the biochemical assay. All authors have reviewed and approved the manuscript.

## Conflict of Interest Statement

The authors declare that the research was conducted in the absence of any commercial or financial relationships that could be construed as a potential conflict of interest.
